# On the Use of a Low-Cost Thermal Sensor to Improve Kinect People Detection in a Mobile Robot

**DOI:** 10.3390/s131114687

**Published:** 2013-10-29

**Authors:** Loreto Susperregi, Basilio Sierra, Modesto Castrillón, Javier Lorenzo, Jose María Martínez-Otzeta, Elena Lazkano

**Affiliations:** 1 Autonomous and Smart Systems Unit, IK4-TEKNIKER, Iaki Goenaga 5, Eibar, Spain; E-Mail: jmmartinez@tekniker.es; 2 Department of Computer Science and Artificial Intelligence, UPV-EHU, Manuel Lardizabal 1, Donostia-San Sebastin, Spain; E-Mails: b.sierra@ehu.es (B.S.); e.lazkano@ehu.es (E.L.); 3 SIANI, Universidad de Las Palmas de Gran Canaria, Juan de Quesada 30, Spain; E-Mails: mcastrillon@iusiani.ulpgc.es (M.C.); jlorenzo@iusiani.ulpgc.es (J.L.)

**Keywords:** sensor fusion, people detection, computer vision, hierarchical classification, mobile robot/platform

## Abstract

Detecting people is a key capability for robots that operate in populated environments. In this paper, we have adopted a hierarchical approach that combines classifiers created using supervised learning in order to identify whether a person is in the view-scope of the robot or not. Our approach makes use of vision, depth and thermal sensors mounted on top of a mobile platform. The set of sensors is set up combining the rich data source offered by a Kinect sensor, which provides vision and depth at low cost, and a thermopile array sensor. Experimental results carried out with a mobile platform in a manufacturing shop floor and in a science museum have shown that the false positive rate achieved using any single cue is drastically reduced. The performance of our algorithm improves other well-known approaches, such as C^4^ and histogram of oriented gradients (HOG).

## 1. Introduction

The deployment of robots as assistants, guides, tutors or social companions in real human environments poses two main challenges: on the one hand, robots must be able to perform tasks in complex, unstructured environments, and on the other hand, robots must interact naturally with humans.

A requirement for natural human-robot interaction is the robot's ability to accurately and robustly detect humans to generate the proper behavior. In this article, the service proposed for the mobile robot is to detect people. This would later allow the robot to decide whether or not to approach the closest person at a given distance with whom to interact. This “engaging” behavior can be useful in potential robot services, such as a tour guide, healthcare or information provider. Once the target person has been chosen, the robot plans a trajectory and navigates to the desired position. To achieve the objectives of our work, the robot must first be able to detect human presence in its vicinity. This must be accomplished without assuming that the person faces the direction of the robot (the robot operates proactively) or wears specific clothing (feasible in an industrial environment, but not in a museum, for instance).

The primary requirement of this research has been to investigate the development of a human detection system based on low-cost sensing devices. Recently, research on sensing components and software led by Microsoft has provided useful results for extracting the human pose and kinematics Shotton *et al.* [1], with the Kinect motion sensor device Kin [2]. Kinect offers visual and depth data at a significantly low cost. While the Kinect is a great innovation for robotics, it has some limitations. First, the depth map is only valid for objects that are further than 80 cm away from the sensing device. A recent study about the resolution of the Kinect by Khoshelham and Elberink [3] proves that for mapping applications, the object must be in the range of 1–3 m in order to reduce the effect of noise and low resolution. Second, the Kinect uses an IRprojector with an IR camera, which means that sunlight could negatively affect it, taking into account that the Sun emits in the IR spectrum. As a consequence, the robot is expected to deal with environments that are highly dynamic, cluttered and frequently subject to illumination changes.

To cope with this, our work is based on the hypothesis that the combination of a Kinect and a thermopile array sensors (low-cost Heimann HTPAthermal sensor Hei [4]) can significantly improve the robustness of human detection. Thermal vision helps to overcome some of the problems related to color vision sensors, since humans have a distinctive thermal profile compared to non-living objects (therefore, human pictures are not considered as positive), and there are no major differences in appearance between different persons in a thermal image. Another advantage is that the sensor data does not depend on light conditions, and people can also be detected in complete darkness. As a drawback, some phantom detections near heat sources, such as industrial machines or radiators, may appear. Therefore, it is a promising research direction to combine the advantages of different sensing sources, because each modality has complementary benefits and drawbacks, as has been shown in other works Bellotto and Hu [5], St-Laurent *et al.* [6], M. Hofmann and Rigoll [7], Johnson and Bajcsy [8], Zin *et al.* [9].

Additional requirements for our application arise from the fact that the low-cost thermal sensor provides a low resolution image and, therefore, does not allow us to build accurate models for detecting people. Moreover, in order to have a high reaction capability, we are looking for solutions that allow parallel processing of all the input data instead of sequentially.

Therefore, the chosen approach is:

- To combine machine learning paradigms with computer vision techniques in order to perform image classification: first, we apply transformations using computer vision techniques, and second, we perform classification using machine learning paradigms.
- To construct a hierarchical classifier combining the three sensor source data (images) to improve person detection accuracy.

We have evaluated the system in two different real scenarios: a manufacturing shop floor, where machines and humans share the space while performing production activities, and a science museum with different elements exposed, people moving around and strong illumination changes, due to weather conditions. Experimental results seem promising considering that the percentage of wrong classifications using only Kinect-based detection algorithms is drastically reduced.

The rest of the paper is organized as follows: In Section II, related work in the area of human detection is presented. We concentrate mainly on work done using machine learning for people detection. Section III describes the proposed approach and Section IV, the experimental evaluation. Section V shows experimental results and Section VI, conclusions and future work.

## 2. Related Work

People detection and tracking systems have been studied extensively because of the increasing demand for advanced robots that must integrate natural human-robot interaction (HRI) capabilities to perform some specific tasks for the humans or in collaboration with them. A complete review on people detection is beyond the scope of this work; extensive work can be found in Schiele [10] and Cielniak [11]. We focus on the recent related work.

To our knowledge, two approaches are commonly used for detecting people on a mobile robot: (1) vision-based techniques; and (2) combining vision with other modalities, normally range sensors, such as laser scanners or sonars, like in Wilhelm *et al.* [12], Scheutz *et al.* [13], Martin *et al.* [14]. Martin *et al.* use a skin color-based detector in a omnidirectional camera and leg profile detectors based on sonar and a laser range-finder to generate specific probability-based hypotheses about detected people and combine these probability distributions by covariance intersection.

The computer vision literature is rich in people detection approaches in color or intensity images. Most approaches focus on a particular feature: the face Hjelmas and Low [15], Yang *et al.* [16], the head, Murphy-Chutorian and Trivedi [17], the upper body or the torso, Kruppa *et al.* [18], Xia *et al.* [19], the entire body, Dalal and Triggs [20], Viola *et al.* [21], Wu *et al.* [22], just the legs, Papageorgiou and Poggio [23] or multimodal approaches that integrate motion information Bellotto and Hu [5]. All methods for detecting and tracking people in color images on a moving platform face similar problems, and their performance depends heavily on the current light conditions, viewing angle, distance to people and variability of the appearance of people in the image.

Apart from cameras, the most common devices used for people tracking are laser sensors. The common aspect in all these approaches is to use distance information to find the human person and then to combine with a visual search for faces or human bodies. Martínez-Otzeta *et al.* [24] present a system for detecting legs and follow a person only with laser readings. A probabilistic model of leg shape is implemented, along with a Kalman filter for robust tracking. This work is extended using thermal information in Susperregi *et al.* [25], using a particle filter to build a people following behavior in a robot. Martinez-Mozos *et al.* [26] address the problem of detecting people using multiple layers of 2D laser range scans. Other implementations, such as Bellotto and Hu [27], also use a combination of face detection and laser-based leg detection and use laser range-finders to detect people as moving objects. The drawbacks of these approaches arise when a person position does not allow one to be distinguished (in lateral position to the robot or near a wall), in scenarios with slim objects (providing leg-like scans). Using only depth images, Zhu and Fujimura [28] proposed a human pose estimation method with Bayesian tracking that is able to detect, label and track body parts. A more promising approach is combining more than one sensory cue. Most existing combined vision-thermal based methods, in St-Laurent *et al.* [6], M. Hofmann and Rigoll [7], Johnson and Bajcsy [8], Zin *et al.* [9], concern non-mobile applications in video monitoring applications and especially for pedestrian detection, where the pose of the camera is fixed. Some works Gundimada *et al.* [29] show the advantages of using thermal images for face detection. They suggest that the fusion of both visible- and thermal-based face recognition methodologies yields better overall performance.

To the authors knowledge, there are few published works on using thermal sensor information to detect humans on mobile robots. Extensive work can be mainly found in the pedestrian detection area Meis *et al.* [30], Li *et al.* [31]. The main reason for the limited number of applications using thermal vision so far is probably the relatively high price of this kind of sensor. Treptow *et al.* [32] and Treptow *et al.* [33] show the use of thermal sensors and grey scale images to detect people in a mobile robot. They build an elliptic contour model and a feature-based model detector to track a person in the thermal image using a particle filter. Guan *et al.* [34] propose a head-shoulder detection based on a stereo-camera fused with the hair and face identified from the thermal-based sensor. Correa *et al.* [35] use face detection based on sate-of-the-art detectors (LBPHistograms) in thermal and visual images for people detection and recognition. These approaches are based on thermal images that require a good resolution in order to build these models, which is not applicable to the low-cost (low-resolution) thermal sensor used in this work.

A drawback of most of these approaches is the sequential integration of the sensory cues; people are firstly detected by thermal information only and are subsequently verified by visual or auditory cues. Thus, any misdetection using the thermal information cannot be recovered using the other sensors.

Most of the above-mentioned approaches have used predefined body model features for the detection of people. Few works considered the application of learning techniques. Arras *et al.* [36] proposed using supervised learning to create a people detector with the most informative features (AdaBoost). Martinez-Mozos *et al.* [26] built classifiers able to detect a particular body part, such as a head, an upper body or a leg, using laser data. These classifiers are learned using a supervised approach based on AdaBoost. The final person detector is composed of a probabilistic combination of the outputs from the different classifiers. Current research in the use of RGB-Dsensors combining color and depth information is extensive; recent works focus on object recognition using color and depth. Lai *et al.* [37] demonstrated in a 300 object dataset that combining color and depth information substantially improves the quality of results. Mozos *et al.* [38] presented a new approach to categorize indoor places using an RGB-D sensor. They built feature vectors combining grey scale images and depth information, which are provided as the input to support vector machines (SVMs) and random forests classifiers, achieving average correct classification rates above 92%. Spinello and Arras Spinello and Arras [39] proposed a new adaptive image and depth data fusion architecture for robust object detection. This architecture allows one to obtain an optimal combination of object detectors, depending on the quality of the sensory cues. This fusion method is applied to people detection, achieving an 87.4% detection rate in their experimental setup.

In a previous work Susperregi *et al.* [40], as the first stage of the present work, a combination of computer vision transformations with machine learning algorithms to use vision and thermal sensor readings to detect if a person is on the view point of the robot was introduced. At that point, the combination was a voting approach; thus, we propose the hierarchical approach in this work.

## 3. Proposed Approach

**Figure 1.**
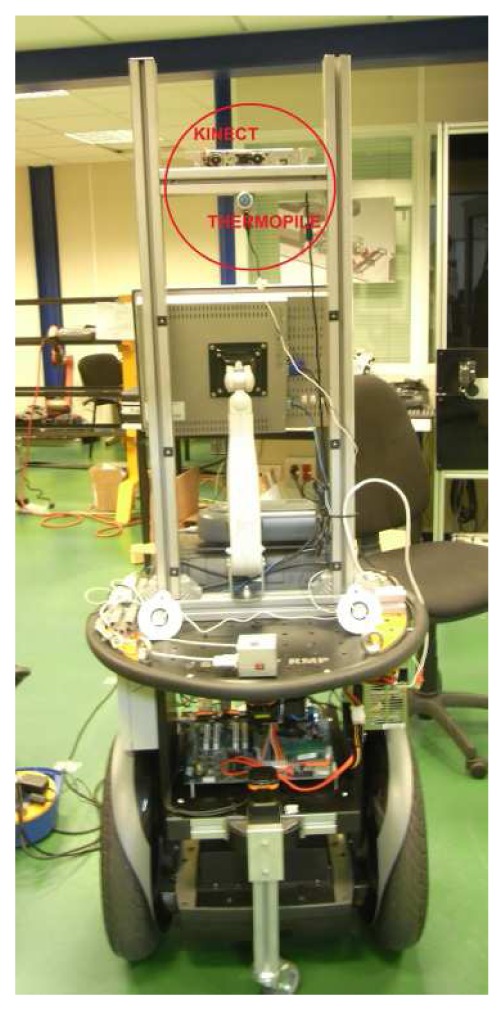
The robotic platform used: a Segway RMP200 provided with the Kinect and the thermal sensor.

We propose a multimodal approach, which is characterized by the processing and filtering of sensory cues. The proposed detection system is based on an HTPA thermal sensor developed by Heimann Hei [4] and a Kinect sensor, mounted on top of an RMPSegway mobile platform, which is shown in Figure 1.

**Figure 2.**
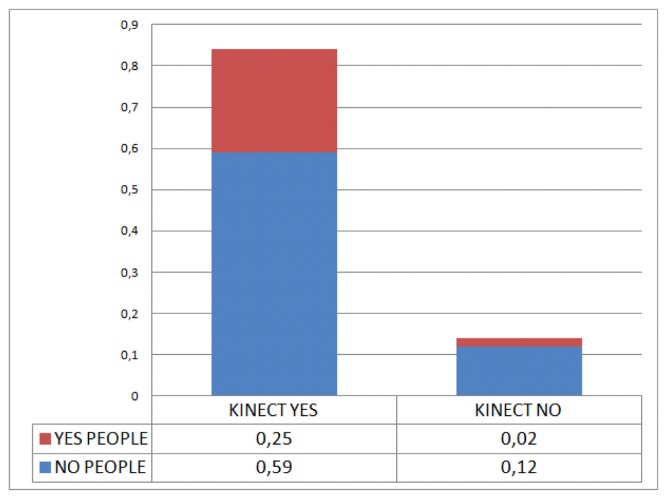
Detection results using Kinect algorithms: IK4-TEKNIKER dataset.

Some preliminary experiments confirm the low people detection ratio achieved by the Kinect sensor-based algorithms Shotton *et al.* [41] in the mobile platform. Figure 2 shows the detection ratio achieved using the dataset collection.The low detection ratio is mainly explained by the algorithms being intended to work for a static camera and not one mounted to a mobile platform.

We aim to apply a new approach to combine machine learning (ML) paradigms with computer vision techniques in order to perform a binary image classification. Our approach is divided into three phases: sensor data prefiltering using computer vision techniques, classification using ML and a combination of classifiers.

**Figure 3.**
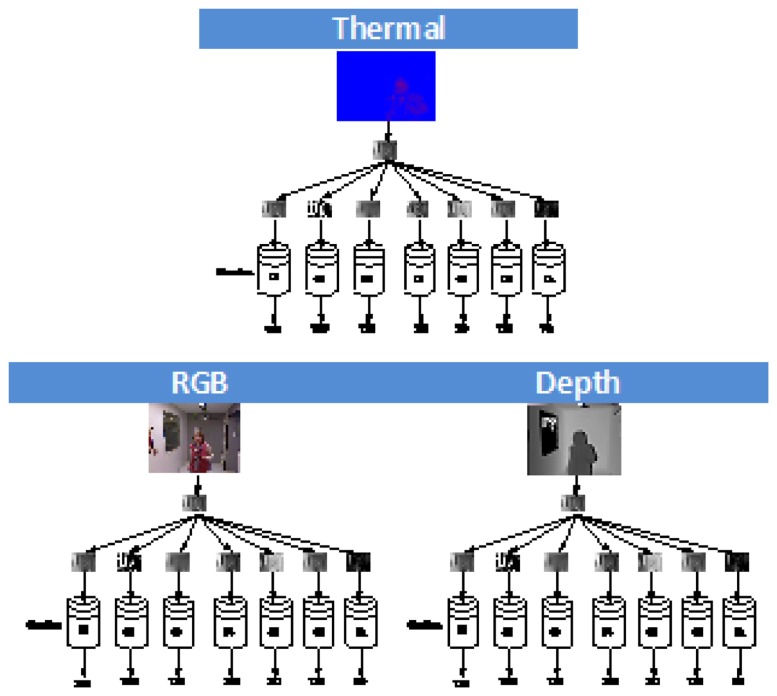
First phase: learning classifiers from three transformed data. Computer vision transformations over the original images are performed to enrich the input database sources.

1. **Computer vision transformations:** In order to have several descriptors of the images, different computer vision transformations over the original images are performed to enrich the input database. The main goal of this phase is to have variability in the features extracted for the same pixel, so that different values are obtained for the same pixel positions; in fact, the information provided by a collection of image transformations is analyzed. As has been mentioned before, we aim to use three input images (color, depth, temperature) to construct a classifier. In this way, and for each of the three data sources, a set of preprocessed images is obtained, one for each of the transformations used.
  To achieve this, we combine some standard image-related algorithms (edge detection, Gaussian filter, binarization, and so on) in order to obtain different image descriptors, and afterwards, we apply some standard machine learning classifiers, taking into account the pixel values of the different modifications of the pictures. Figure 3 shows an example, in which some of the transformations are used. From the original training database collected, a new training database is obtained for each of the computer vision transformation used, summing up a total of 24 databases for each sensor.
2. **In the classification phase**, the system learns a classifier from a hand-labeled dataset of images (the above-mentioned original and transformed images). Five well-known ML supervised classification algorithms with completely different approaches to learning and a long tradition in classification tasks are used: IB1, Naive-Bayes, Bayesian Network, C4.5 and SVM.
3. **Fusion phase:** Finally, the goal of our fusion process is to maximize the benefits of each modality by intelligently fusing their information and by overcoming the limitations of each modality alone.

### 3.1. Data Acquisition and Transformation

As stated before, three kinds of data sources are used coming from the Kinect sensor and the thermopile array.

**Figure 4.**
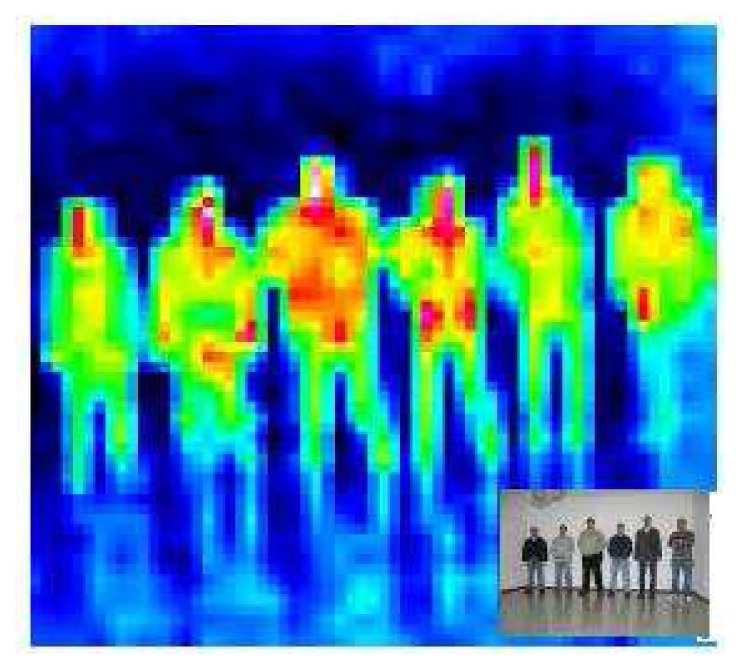
HTPAthermopile image sample and a miniature of its corresponding RGB image.

1. The HTPA allows for the measurement of the temperature distribution of the environment, where very high resolutions are not necessary, such as person detection, surveillance of temperature critical surfaces, hotspot or fire detection, energy management and security applications. The thermopile array can detect infrared radiation; we convert this information into an image in which each pixel corresponds to a temperature value. The sensor only offers a 32 × 31 image, which allows for a rough resolution of the environment temperature, as is shown in Figure 4. People present a thermal profile different from their surrounding environment. The temperature detected in the pixel corresponding to a person is usually around 37 Celsius degrees, with some tendency of being a bit lower, due to the presence of hair or clothes over the skin.
  The benefits of this technology are the very small power consumption, as well as the high sensitivity of the system.
2. Kinect provides depth data, which we transform into depth images; it uses near-infrared light to illuminate the subject, and the sensor chip measures the disparity between the information received by the two IR sensors. It provides a 640 × 480 distance (depth) map in real time (30 fps).
3. In addition to the depth sensor, the Kinect also provides a traditional 640 × 480 RGB image.

In order to calibrate both sensors' data, the following have to be considered:

1. Horizontal FOV: It is known that the Kinect horizontal FOV (RGB) is 62.7 degrees for 640 pixels, while the thermopile horizontal FOV is 38 degrees for 32 pixels. As the thermopile center is vertically aligned with the Kinect center, it covers the 387.8788 (640 * 38 / 62.7) central horizontal pixels of the Kinect RGB image; so, it covers the pixels in the range ([126.06–513.94]).
2. Vertical FOV: In the vertical range, following the same proportions as in the horizontal range, the thermopile covers 375.7603 (12.1213 * 31) Kinect RGB vertical pixels, which means that, if the sensors' centers were to be in the same place, the thermopile would cover the pixels in the range (52.11985, 427.88015). As the thermopile is located over the Kinect, the pixels in the range (0, 370) are covered.

#### 3.1.1. Computer Vision Transformations

**Figure 5.**
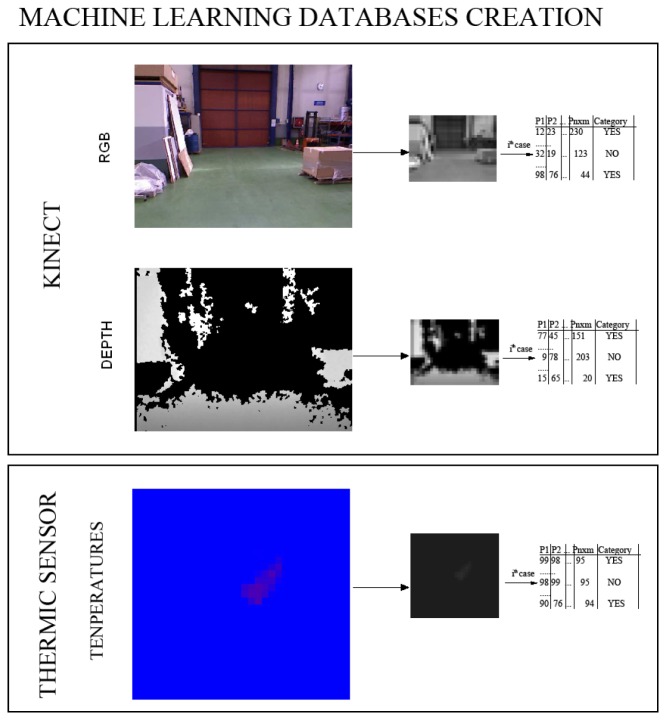
Image preprocessing and training database creation from a hand-labeled original dataset and transformed images.

The three data sources acquired in parallel (image, distance, temperature) are used to build a classifier, whose goal is to identify whether a person is in the view-scope of the robot or not. Figure 5 shows an example of the three different images obtained; each original image is scaled to 32 × 24 and converted to gray scale. The value of each pixel position in the matrix is considered as a predictor variable within the machine learning database construction, summing up *n* × *m* features, *m* being the column number and *n*, the row number in the image. Each image corresponds to a single row in the generated database.

**Figure 6.**
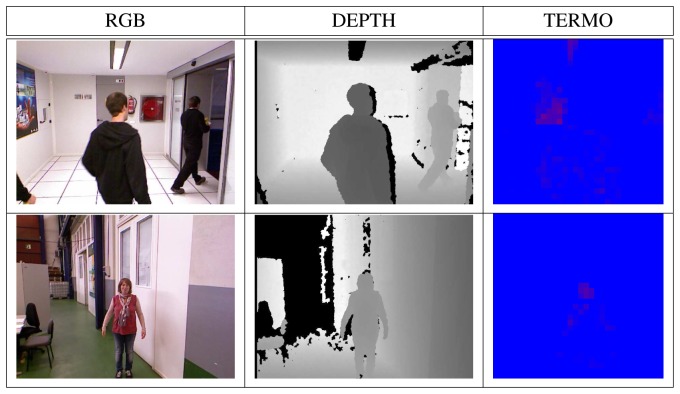

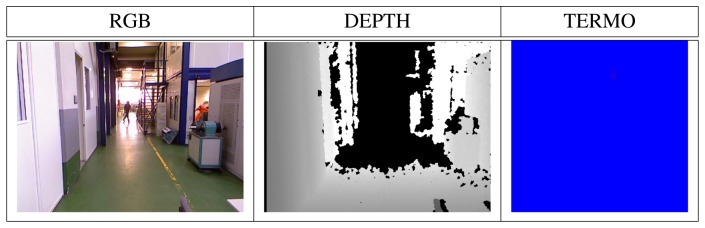
Positive examples in the three data sources (intensity, depth, thermal) with people with different sizes and positions.

In order to have different descriptors of the images, modifications over the original images are performed. The databases contain people in different pose and scales in order to introduce variability and to provide robustness under translations, rotation or scale changes; see Figure 6.

We have selected some of the most common transformations, in order to show the benefits of the proposed approach, making use of simple algorithms. Table 1 presents the collection of transformations used, as well as a brief description of each one of them. It is worth pointing out the fact that any other CVtransformation could be used apart from the selected ones.

### 3.2. Machine Learning Classifiers

Five well-known ML supervised classification algorithms with completely different learning approaches and a long tradition in different classification tasks are used: IB1, Naive-Bayes, Bayesian Network, C4.5 and SVM. Later, the goal of our fusion process is to maximize the benefits of each modality by intelligently fusing their information and by overcoming the limitations of each modality alone.

1. IB1
  The IB1 Aha *et al.* [42] is a case-based, nearest-neighbor classifier. To classify a new test sample, all training instances are stored, and the nearest training instance regarding the test instance is found; its class is retrieved to predict this as the class of the test instance.
2. Naive-Bayes
  The Naive-Bayes (NB) rule Cestnik [43] uses the Bayes theorem to predict the class for each case, assuming that the predictive attributes are independent given the category. To classify a new sample characterized by *d* attributes, **X** = (*X*_1_, *X*_2_, …, *X_d_*), the NB classifier applies the following rule:
  $$c_{N-B}=\arg\underset{c_{j}\in C}{\max}p(c_{j})\prod_{i=1}^{d}p(x_{i}|c_{j})$$
  where *c_N_*_−_*_B_* denotes the class label predicted by the Naive-Bayes classifier and the possible l classes of the problem *C* = {*c*_1_, …, *c_l_*}.
3. Bayesian Networks
  A Bayesian network, belief network or directed acyclic graphical model is a probabilistic graphical model that represents a set of random variables and their conditional independencies via a directed acyclic graph (DAG). For example, a Bayesian network could represent the probabilistic relationships between diseases and symptoms. Given symptoms, the network can be used to compute the probabilities of the presence of various diseases. There are many classifiers based on the probability theory. Most of them use ideas from the Bayes theorem and try to obtain the class whose *a posteriori* probability is greater given the values of the predictor variables of the case to be classified. In other words, probabilistic classifiers give to the new case the most likely class for the values its variables have. In this paper, we have used Bayesian Networks as classification models, proposed by Sierra *et al.* [44].
4. C4.5
  The C4.5 Quinlan [45] represents a classification model by a decision tree. It is run with the default values of its parameters. The tree is constructed in a top-down way, dividing the training set and beginning with the selection of the best variable in the root of the tree. The selection of the best feature is performed by the maximization of a splitting criterion, which is based on an informatics theoretic approach. For each continuous attribute, a threshold that maximizes the splitting criterion is found by sorting the cases of the dataset on their values of the attribute: every pair of adjacent values suggests a threshold in their midpoint, and the threshold that yields the best value of the splitting criterion is selected. A descendant of the root node is then created for each possible value of the selected feature, and the training cases are sorted to the appropriate descendant node. The entire process is then recursively repeated using the training cases associated with each descendant node to select the best feature to test at that point in the tree. The process stops at each node of the tree when all cases in that point of the tree belong to the same category or the best split of the node does not surpass a fixed chi-square significancy threshold. Then, the tree is simplified by a pruning mechanism to avoid overspecialization.
5. Support Vector Machines (SVMs)
  SVMs are a set of related supervised learning methods used for classification and regression. Considering a two-class problem where the input data of each class is viewed as an n-dimensional vector, an SVM will construct a separating hyperplane in that space, one which maximizes the margin between the two datasets. To calculate the margin, two parallel hyperplanes are constructed, one on each side of the separating hyperplane, which are “pushed up against” the two datasets. Intuitively, a good separation is achieved by the hyperplane that has the largest distance to the neighboring data points of both classes, since, in general, the larger the margin, the lower the generalization error of the classifier Meyer *et al.* [46].

### 3.3. Combination of Classifiers/Sensors

In order to finally classify the targets as containing a human or not, the estimation of the RGB-D-based classifiers is combined with the estimation of the temperature-based classifier. After building the individual classifiers (5 × 24 = 120 for each cue), the aim is to combine the output of different classifiers to obtain a more robust final people detector.

**Figure 7.**
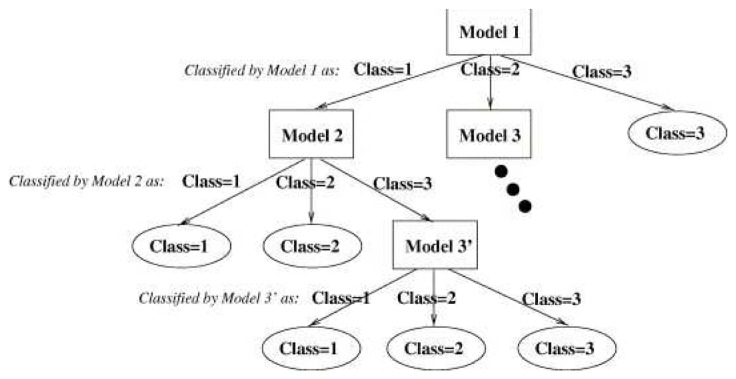
Hierarchical classifier schemata.

The last step is to combine the results of the best three classifier obtained, one for each input image type (intensity, depth, temperature). To achieve this, we use the hierarchical classifier approach by Martínez-Otzeta *et al.* [47], Sierra *et al.* [48] in which the decision of each of the three single classifiers is combined in a tree mode in order to increase the overall accuracy. Figure 7 shows the typical approach used to perform a classification with this multiclassifier approach.

We have used this multiclassifier to combine the different sensors, selecting, at each step, the classifier learned in this type of image that increases the accuracy the most. One of the reasons we do that is mainly related to the computational load: using only three classifiers, one for each sensor, we can make the needed preprocess in parallel, obtaining a faster answer. In this way, the resulting model can operate in real time, a mandatory feature for the task to be accomplished.

## 4. Experimental Evaluation

In this section, we present the experimental evaluation of our approach carried out using data collected with a mobile robot in two scenarios: a manufacturing plant and a science museum. The results obtained using other approaches are relevant to assess whether the method presented is competitive enough and, therefore, worth continuing in the proposed direction. We have compared our approach with other relevant approaches in people detection, such as the histogram of oriented gradients (HOG) and the *C*^4^ algorithm.

HOGs are a kind of feature descriptors, which compute the number of occurrences of a gradient orientation (histogram) in portions of an image. In their seminal work, Dalal and Triggs [49] focused on the problem of pedestrian detection in static images, though the technique could be applied to other domains, as well.

A more recent people detection algorithm, *C*^4^ Wu *et al.* [22], detects humans using contour cues, a cascade classifier and the CENTRIST visual descriptor. The authors claim that *C*^4^ has shown a competitive recognition rate when compared to HOG; the algorithm uses contour information for human detection, and it is extremely fast. We have decided to collect the recognition rate this algorithm offers in the databases with which we are working.

It is worth mentioning that we are evaluating only the detection accuracy, although these algorithms can also provide people tracking capabilities.

### 4.1. Experimental Data

To obtain positive and negative examples in both scenarios, the robot was operated in two unconstrained indoor environments (the manufacturing plant and the science museum). At the same time, image data was collected with a frequency of 1 Hz. The images were hand-labeled as positive examples if people were visually detected in the image and as negative examples, otherwise.

#### 4.1.1. Dataset in Manufacturing Scenario

**Figure 8.**
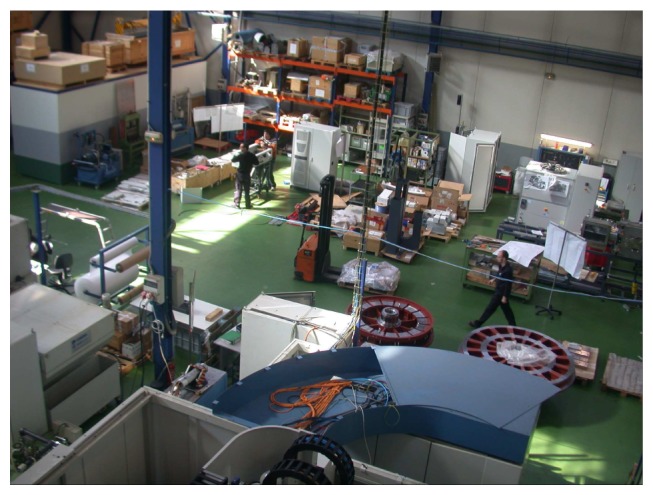
Manufacturing plant at IK4-TEKNIKER.

The manufacturing plant located at IK4-TEKNIKER is a real manufacturing shop floor, where machines and humans share the space during production activities. The shop floor, as seen in Figure 8, can be characterized as an industrial environment, with high ceilings, fluorescent light bulbs, high windows, *etc.* The lighting conditions change from one day to another and even in different locations along the path covered by the robot.

**Figure 9.**
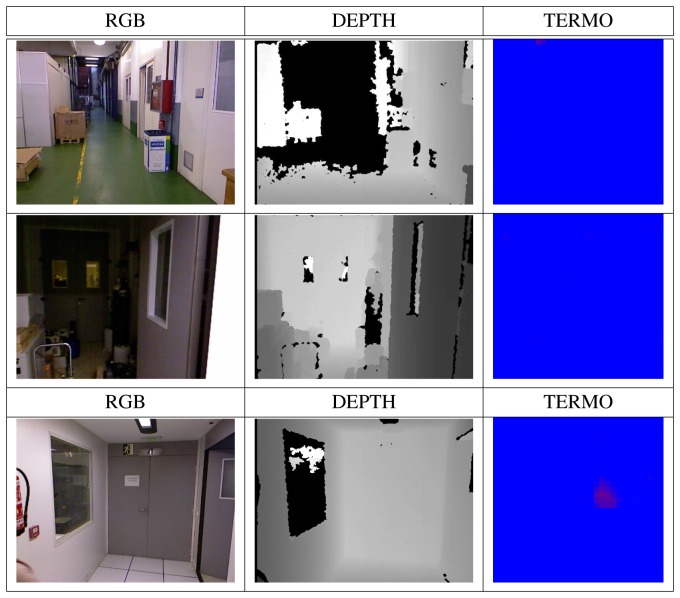
Negative examples in the three data sources (intensity, depth, thermal), with different elements in the environment.

The dataset is composed of 1,064 samples. The input to the supervised algorithms is composed of 301 positive and 763 negative examples. The set of positive examples contains people at different positions and dressed with different clothing in a typical manufacturing environment. The set of negative examples is composed of images with no human presence and containing other objects, such as machines, tables, chairs, walls, *etc.*Figures 6 and 9 show some database samples. It has to be noticed that the thermal images shown in the third column of the figures do not always discriminate the presence of a person, due to the existence of hot elements in the plant (which could produce false positives) or, on the contrary, the clothing of a given person who is not looking at the robot, which could give a false negative case.

#### 4.1.2. Dataset in Science Museum: EUREKA!

**Figure 10.**
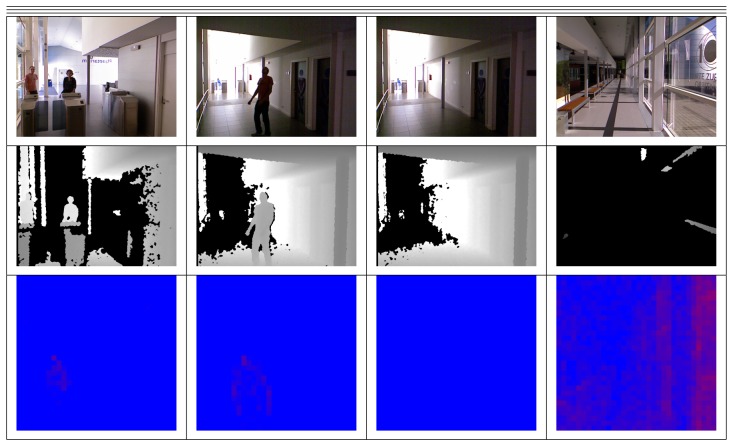
Images from the Eureka! Science Museum in the three data sources (intensity, depth, thermal), where different issues relevant to the problem are represented. From the left: many persons, people and objects with similar silhouettes and Sun incidence in corridors.

The EUREKA! Science Museum is the second scenario in which the robot has been evaluated to identify the presence of people. Figure 10 shows some images taken by the robot; the lighting conditions also affect the image treatment, as there are crystal corridors in the museum. In addition, there are some aesthetic elements that can be detected as persons.

This dataset is composed of 619 samples (392 positive and 227 negative). The positive/negative distribution is different compared with the previous dataset, in order to better appreciate the generalization capabilities of the approaches used.

#### 4.1.3. Experimental Methodology

These are the steps of the experimental phase:

1. Collect a database of images that contains three data types that are captured by the two sensors: 640 × 480 depth map, 640 × 480 RGB image and 32 × 31 thermopile array.
2. Reduce the image sizes from 640 × 480 pixels to 32 × 24 pixels, and convert color images to gray-scale ones.
3. For each image, apply 23 computer vision transformations (see Table 1), obtaining 23 transformed images for each image type. Thus, we have 24 datasets for each image type.
4. Build 120 classifiers, applying 5 machine learning algorithms for each image type training dataset (5 × 24) and using 10-fold cross-validation Stone [50].
5. Apply 10-fold cross-validation using 5 different classifiers for each of the previous databases, summing up a total of 3 × 24 × 5 = 360 validations.
6. In each node of the hierarchical multiclassifier, select the classifier with the lowest error rate.
7. Make a final decision combining the results of the classifiers.

#### 4.1.4. Metrics

The performance of the people detection system is evaluated in terms of detection rates (accuracy) and false positives/negatives. True positives (TPs) are the people images detected from the ground truth. False negatives (FNs) are the people images not detected from the ground truth, and false positives (FPs) are images detected as people that do not appear in the ground truth. The performance evaluation is done with the following score:

(1) $$\text{Accuracy}=\frac{TP+TN}{TP+FP+TN+FN}$$

where *TP*, *TN*, *FP* and *FN* are, respectively, true positives, true negatives, false positives and false negatives.

**Figure 11.**
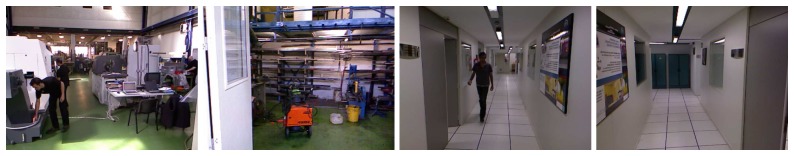
Respectively, a true positive, false positive, false negative and true negative example using our approach.

**Figure 12.**
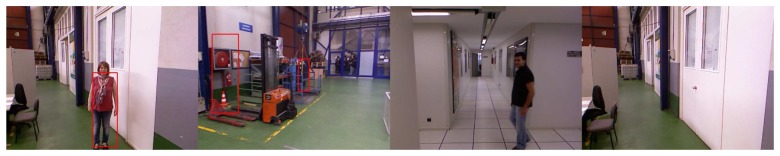
Respectively, a true positive, false positive, false negative and true negative example using the *C*^4^ approach.

Figures 11 and 12 show some examples of the kind of results obtained by our approach and the *C*^4^ method, respectively.

## 5. Results

### 5.1. IK4-TEKNIKER

In order to make a fast classification (a real-time response is expected), we first transform, as mentioned above, the color images to gray-scale 32 × 24, and reduce, as well, the size of the infrared images to a 32 × 24 size matrix. Hence, we have to deal with 768 predictor variables, instead of 307, 200 × (three colors) of the original images taken by the Kinect camera.

First of all, we have used the five classifiers using the reduced original databases (32×24 for intensity and depth, 31 × 31 for thermal pictures). Table 2 shows the 10-fold cross-validation accuracy obtained using the input images without transformation. The best result is 92.11% for the thermal image original database, using SVM as the classifier. The real-time Kinect's algorithms accuracy for the same images was quite poor (37.50%), as the robot was moving. As a matter of fact, that has been the main motivation of the presented research.

The same accuracy validation process has been applied to each image transformation on each image format. Table 3 shows the results obtained by each classifier on the resulting transformed 23-image databases. The best result is obtained by the C4.5 classifier after transforming the images using Transformation 7 (Gaussian one). This classifier is selected as the best intensity-based classifier to be combined with the other two best classifiers.

After performing the validation over the depth images, the results shown in Table 4 are obtained. The best result is obtained again by the C4.5 classifier after transforming the images using Transformation 7 (Gaussian one), with a 92.82 accuracy. This classifier is selected as the distance image (depth) one to take part in the final combination.

Finally, the classifiers are applied to the thermal images, obtaining the results shown in Table 5. In this case, we obtain the best result (93.52) for the SVM classifier, and for two of the used transformations (Transf.8 (Lat) and Transf. 9 (Linear-stretch)). Moreover, the obtained results are identical for both paradigms, so any of them can be used in the final combination, obtaining indistinct results.

### 5.2. Science Museum

The same process has been applied to the science museum dataset. Table 6 shows the results obtained with the original intensity images. The best result (87.08) is obtained for the *intensity* images using the K-NNalgorithm. For *Depth* data, the best result (79.16) is obtained by means of a Bayesian Network classifier, while for the *Thermal* data, the K-NN classifier obtains 80.45 as the best result.

When the transformations are applied, an increment in the obtained accuracy is achieved for all the data sources and all the classifiers used. The obtained results are shown in Table 7; once again, the best result is obtained for the *intensity* images using the K-NN algorithm (90.79), using the sixth transformation; using *Depth* data, the best result (80.94) is obtained by the Bayesian Network classifier after the 12th CV transformation, while for the *Thermal* data, the K-NN classifier obtains 84.49 as the best result, combined with the sixth transformation.

#### 5.2.1. Bayesian Network Structure

Bayesian Networks are paradigms used to represent the joint probability of a set of (discrete) variables. As stated before, they can be used as classifiers in a supervised classification problem, and in this case, the existence of a variable of interest has to be taken into account: that corresponding to the class. It is worth mentioning that the Bayesian Network classifier takes as predictor variables the pixels of the images.

As can be seen, the Bayesian Network structure can be very complex, and it is necessary to put emphasis on those nodes belonging to the so-called Markov Blanket of the *Class* node, composed of its parents, its direct descendants and the parents of those descendants.

### 5.3. Hierarchical Multiclassifier

**Figure 13.**
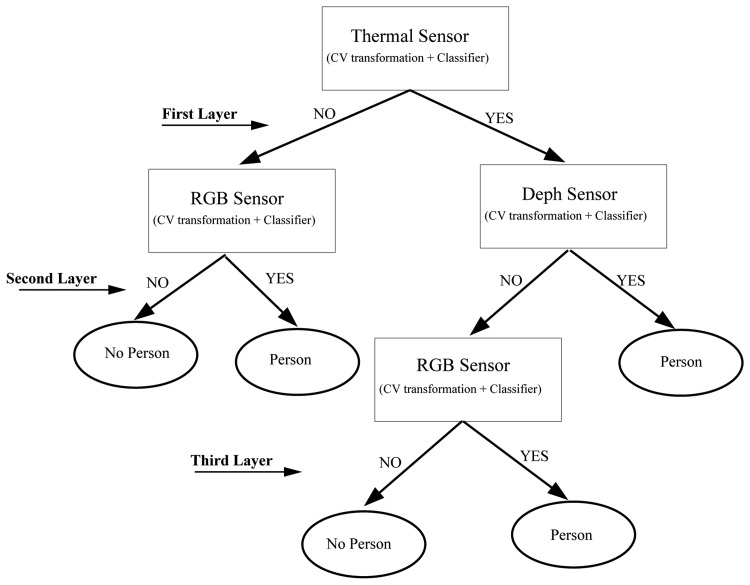
Example of a hierarchical classifier.

The last step is to combine the three best classifiers obtained, one for each sensor. This has been done using a hierarchical multiclassifier Martínez-Otzeta *et al.* [47]. A tree-shaped classifier is constructed. The decision of each node is performed by a single classifier, learned for the corresponding data. We have decided to specialize each node for one sensor data type (among the three used), and thus, this sensor type data is not to be used in the nodes below. Figure 13 shows an example of the multiclassifier used. As can be seen, in this example, the top node (also known as the root of the tree) is devoted to the thermal sensor data. For this data, the best (CV Transformation, Classifier) pair is selected. To continue with the classifier construction, for each of the arcs of the tree, a database is needed in order to learn the corresponding classifier, which aims to correct some of the errors made by its top node model.

To do this, using a 10-fold cross-validation, the cases classified as *No person* are selected, and the corresponding cases of the other two sensors are used to obtain the best CV transformation and the combination of classifiers. The example assumes that the best results are obtained using the intensity data for some CV transformation and classifier.

The construction of the multiclassifier continues in this manner, a new case selection is performed for the images classified as containing persons (right side) and for the images classified as not containing persons (left side). In this example, when images are labeled as *No person* by the root node (thermal data), trying to outperform, through the depth data, the results obtained using the intensity data to correct some errors made by the thermal data, no improvements are obtained. Thus, the depth sensor is not used on the right side of the tree, *i.e.*, the results given by the intensity based classifier are the final answer of the multiclassifier. On the left side of this example, the best results are obtained using data from the depth sensor (with a corresponding transformation and classifier CV); a new experiment is performed to correct some errors, and these are corrected using the intensity-based classifier. Therefore, to classify a new case, when the paradigm based on the thermal sensor classifies as *Person*, but the depth sensor classifier gives *No person*, the RGB sensor classifier sets the final decision.

Table 8 shows the results obtained using each cue as the root node. As can be seen, in the IK4-TEKNIKER database, the best obtained accuracy is 96.74%, using the thermal sensor data to construct the root node classifier. It significantly improves the result of the best previous classifiers (93.52) for the thermal images. The best classifier obtained for the EUREKA! database has a 94.99% of well-classified cases, which outperforms as well the best previous result (90.79) as well.

### 5.4. Results Obtained by HOG

To obtain HOG-based proposals, we use the GPUimplementation in OpenCV. The detector can process 640 × 480 images in 5–10 Hz. The full body detections are obtained from the model trained on our databases.

Table 9 shows the results obtained by the *HOG* algorithm. The results indicate that the best accuracy is obtained for the intensity images provided by Kinect (72.24%), followed by the application to the depth cue, an approach similar to HODSpinello Spinello and Arras [51] with a false negative rate of 72.78%. With the thermal images, 99.87% of images are classified as not containing any person.

### 5.5. Results Obtained by C^4^

Table 10 shows the results obtained by the *C*^4^ algorithm; original images have been used, as the results using the reduced size are very poor.

As can be seen, the best accuracy is obtained for the intensity images provided by Kinect (77.00%), with a false negative rate of 17.80%. With the thermal images, as the size is too small (32 × 31) the method is not adequate and classifies all the images as not containing any person.

## 6. Conclusions and Future Work

This paper has presented a people detection system for mobile robots using an RGB-D and thermal sensor fusion. The system uses a hierarchical classifier combination of computer vision and machine learning paradigms to decide if a person is in the view-scope of the robot or not. This approach has been designed to manage three kinds of input images, color, depth and temperature, to detect people. We have provided an experimental evaluation of its performance. On the one hand, we have shown that the person detection accuracy is improved, while decreasing the FPR by cooperatively classifying the feature matrix computed from the input data. On the other hand, experimental results have shown that our approach performs well, comparing with state-of-the-art people detection algorithms in the datasets used. This work serves as an introduction to the potential of multi-sensor fusion in the domain of people detection in mobile platforms.

In the near future, we envisage:

- evaluating the system in other scenarios, comparing with current state-of-the-art approaches.
- using input feature selection that is invariant under translations or changes in scale; improving results, adding more sophisticated transformation and applying other computer vision paradigms, such as key point detectors (**SIFT**, *etc.*) or geometrical shape constraints (wavelets, *etc.*).
- extending the detection algorithms in order to distinguish single and multiple people in the image.
- improving people detection by: using other detection algorithms (**HOG**, *C*^4^) and motion information as the input; using other stacking combination approaches for classifiers.
- developing trackers combining/fusing visual cues using particle filter strategies, including face recognition, in order to track people or gestures; integrating with robot navigation planning ability to explicitly consider humans in the loop during robot movement.
- optimizing implementations in order to achieve high detection speeds to use in real-time applications.

## Figures and Tables

**Table 1.** Image transformation description.

***Transform***	**Command**	**Effect**
*Transf.1*	Convolve	Apply a convolution kernel to the image
*Transf. 2*	Despeckle	Reduce the speckles within an image
*Transf. 3*	Edge	Apply a filter to detect edges in the image
*Transf. 4*	Enhance	Apply a digital filter to enhance a noisy image
*Transf. 5*	Equalize	Perform histogram equalization to an image
*Transf. 6*	Gamma	Perform a gamma correction
*Transf. 7*	Gaussian	Reduce image noise and reduce detail levels
*Transf. 8*	Lat	Local adaptive thresholding
*Transf. 9*	Linear-Str.	Linear with saturation histogram stretch
*Transf. 10*	Median	Apply a median filter to the image
*Transf. 11*	Modulate	Vary the brightness, saturation and hue
*Transf. 12*	Negate	Replace each pixel with its complementary color
*Transf. 13*	Radial-blur	Radial blur the image
*Transf. 14*	Raise	Lighten/darken image edges to create a 3D effect
*Transf. 15*	Selective-blur	Selectively blur pixels within a contrast threshold
*Transf. 16*	Shade	Shade the image using a distant light source
*Transf. 17*	Sharpen	Sharpen the image
*Transf. 18*	Shave	Shave pixels from the image edges
*Transf. 19*	Sigmoidal	Increase the contrast
*Transf. 20*	Transform	Affine transform image
*Transf. 21*	Trim	Trim image edges
*Transf. 22*	Unsharp	Sharpen the image
*Transf. 23*	Wave	Alter an image along a sine wave

**Table 2.** 10-fold cross-validation accuracy percentage obtained for each classifier using IK4-TEKNIKER original images. NB, Naive-Bayes; SVM, support vector machine.

***Data source***	**BN**	**NB**	**C4.5**	**K-NN**	**SVM**
*Visual*	89.20	71.74	82.63	90.89	85.35
*Depth*	86.29	68.64	83.29	90.89	84.04
*Thermal*	89.67	86.10	87.79	91.74	**92.11**

**Table 3.** IK4-TEKNIKER intensity images: 10-fold cross-validation accuracy percentage obtained for each classifier using each of the proposed transformations.

***Images***	**BN**	**NB**	**C4.5**	**K-NN**	**SVM**
Transf. 1	89.20	71.74	90.89	82.63	85.35
Transf. 2	87.89	72.30	90.99	84.41	86.29
Transf. 3	83.19	74.84	87.98	75.87	81.41
Transf. 4	88.92	71.92	90.89	82.44	86.20
Transf. 5	86.76	71.64	89.77	80.47	80.66
Transf. 6	87.98	71.36	90.89	83.29	86.29
Transf. 7	87.79	64.79	**91.83**	85.92	84.79
Transf. 8	76.81	78.03	85.07	71.36	76.90
Transf. 9	88.54	73.90	91.17	81.31	84.98
Transf. 10	87.98	69.48	90.70	82.82	84.69
Transf. 11	85.54	72.96	91.55	82.07	85.26
Transf. 12	88.92	71.74	90.89	82.63	85.35
Transf. 13	88.73	68.64	90.99	82.63	85.45
Transf. 14	88.83	71.74	90.89	83.76	85.54
Transf. 15	89.20	71.74	90.89	82.63	85.35
Transf. 16	83.85	75.12	86.38	77.93	81.78
Transf. 17	89.77	71.46	90.23	83.00	82.44
Transf. 18	88.73	71.55	90.61	82.35	85.35
Transf. 19	88.17	70.61	91.46	82.82	86.10
Transf. 20	89.11	70.99	90.80	82.63	84.98
Transf. 21	89.20	71.74	90.89	82.63	85.35
Transf. 22	88.83	71.36	90.33	82.35	82.72
Transf. 23	88.73	72.30	90.80	83.85	85.82

**Table 4.** IK4-TEKNIKER depth images: 10-fold cross-validation accuracy percentage obtained for each classifier using each of the proposed transformations.

***Distances***	**BN**	**NB**	**C4.5**	**K-NN**	**SVM**
Transf. 1	86.29	68.64	90.89	83.29	84.04
Transf. 2	86.38	68.45	91.27	83.38	82.91
Transf. 3	83.66	78.87	87.23	78.97	81.60
Transf. 4	86.10	68.54	90.89	82.91	83.29
Transf. 5	85.35	70.80	90.89	80.38	81.97
Transf. 6	86.38	70.33	90.61	82.25	83.76
Transf. 7	85.92	66.95	**92.86**	85.26	84.23
Transf. 8	83.19	73.62	84.04	73.15	78.40
Transf. 9	85.26	67.70	90.33	83.00	83.19
Transf. 10	85.54	68.92	92.30	85.16	85.35
Transf. 11	84.69	68.26	90.99	81.50	82.35
Transf. 12	86.67	68.64	90.89	83.38	84.04
Transf. 13	85.35	68.08	92.21	82.54	83.29
Transf. 14	86.57	68.73	90.89	83.76	84.13
Transf. 15	86.29	68.64	90.89	83.29	84.04
Transf. 16	83.66	78.69	87.14	80.38	85.35
Transf. 17	85.63	71.27	90.52	82.25	81.50
Transf. 18	85.63	66.20	89.77	82.72	82.54
Transf. 19	86.48	70.05	90.89	83.85	83.94
Transf. 20	86.67	69.01	90.70	83.29	83.85
Transf. 21	85.45	70.33	91.36	83.29	82.82
Transf. 22	85.73	71.08	90.42	81.78	81.60
Transf. 23	85.92	68.64	91.27	80.47	83.10

**Table 5.** IK4-TEKNIKER thermal images: 10-fold cross-validation accuracy percentage obtained for each classifier using each of the proposed transformations.

***Thermal images***	**BN**	**NB**	**C4.5**	**K-NN**	**SVM**
Transf. 1	89.67	86.10	91.74	87.79	92.11
Transf. 2	90.99	84.32	92.39	91.46	92.58
Transf. 3	89.30	86.67	90.80	86.29	92.39
Transf. 4	89.11	83.85	92.49	89.39	90.33
Transf. 5	85.73	84.60	92.77	90.33	85.63
Transf. 6	89.67	85.92	91.74	87.79	91.83
Transf. 7	86.57	82.16	89.67	87.79	89.95
Transf. 8	89.11	85.92	91.64	84.04	**93.52**
Transf. 9	90.80	88.08	92.39	87.89	**93.52**
Transf. 10	84.98	81.97	86.29	80.56	85.63
Transf. 11	71.74	71.74	71.74	71.74	71.74
Transf. 12	89.77	85.63	91.74	87.79	92.11
Transf. 13	90.05	84.69	92.77	90.14	91.08
Transf. 14	89.11	86.01	91.08	87.89	91.83
Transf. 15	89.67	86.10	91.74	87.79	92.11
Transf. 16	89.48	86.85	91.17	90.33	89.95
Transf. 17	89.67	87.23	91.74	87.04	90.99
Transf. 18	89.11	85.63	91.55	85.63	89.86
Transf. 19	89.67	85.07	91.83	87.79	91.83
Transf. 20	89.77	86.01	91.74	87.79	92.68
Transf. 21	83.57	47.89	84.41	82.54	72.02
Transf. 22	89.77	85.82	91.92	87.79	91.17
Transf. 23	90.05	85.45	92.02	90.33	91.27

**Table 6.** Best 10-fold cross-validation accuracy percentage obtained for each classifier using the EUREKA! original images.

***Data source***	**BN**	**NB**	**C4.5**	**K-NN**	**SVM**
*Intensity*	81.74	59.94	79.64	**87.08**	83.84
*Depth*	79.16	63.00	72.54	74.47	72.86
*Thermal*	79.97	60.01	78.03	80.45	77.38

**Table 7.** EUREKA!: best 10-fold cross-validation accuracy percentage obtained for each classifier using transformed images. The corresponding transformation is indicated.

***Data source***	**BN**	**NB**	**C4.5**	**K-NN**	**SVM**
Intensity	87.24 Transf.4	73.02 Transf.15	83.52 Transf.6	**90.79** Transf.6	85.14 Transf.5
Depth	80.94 Transf.12	65.75 Transf.4	75.44 Transf.17	78.03 Transf.6	75.61 Transf.15
Thermal	82.39 Transf.4	74.34 Transf.2	80.61 Transf.3	84.49 Transf.6	79.16 Transf.8

**Table 8.** Hierarchical multiclassifier: 10-fold cross-validation accuracy percentage obtained selecting each sensor image as the root node.

***Database***	**Source**	**First Layer**	**Second Layer**	**Third Layer**
IK4-Tekniker	Visual	91.83	94.55	–
	Depth	92.86	95.68	–
	Thermal	93.52	94.55	**96.74**

Eureka!	Visual	90.79	**94.99**	–
	Depth	80.94	91.11	–
	Thermal	84.49	88.85	92.33

**Table 9.** Results obtained by the histogram of oriented gradients (*HOG*) approach, compared with those obtained with the proposed approach. TP, true positive; FP, false positive; TN, true negative; FN, false negative.

***Approach***	**Accuracy**	**TP**	**FP**	**TN**	**FN**	**Prec.**	**Recall**
*Intensity (HOG)*	72.24	63.79	36.21	27.22	47.25	0.6379	0.5745
*Depth (HOD)*	72.02	63.79	36.21	52.75	72.78	0.6379	0.4671
*Thermal (HOG)*	51.93	1.13	98.67	0.13	99.87	0.0113	0.0112

*Our Approach*	96.74	95.36	4.64	98.12	1.88	0.9536	0.9807

**Table 10.** Results obtained by the *C*^4^ approach, compared with those obtained with the proposed approach.

***Approach***	**Accuracy**	**TP**	**FP**	**TN**	**FN**	**Prec.**	**Recall**
*Intensity (C*^4^*)*	77.00	63.79	36.21	82.20	17.80	0.6379	0.7818
*Depth (C*^4^*)*	72.39	51.16	48.84	80.76	19.24	0.5116	0.7267
*Thermal (C*^4^*)*	71.17	0.00	1.00	1.00	0.00	0	NAN

*Our Approach*	96.74	95.36	4.64	98.12	1.88	0.9536	0.9807
